# Glucagon-like peptide-1: a multi-faceted anti-inflammatory agent

**DOI:** 10.3389/fimmu.2023.1148209

**Published:** 2023-05-17

**Authors:** Syed Faizan Mehdi, Suma Pusapati, Muhammad Saad Anwar, Durga Lohana, Parkash Kumar, Savitri Aninditha Nandula, Fatima Kausar Nawaz, Kevin Tracey, Huan Yang, Derek LeRoith, Michael J. Brownstein, Jesse Roth

**Affiliations:** ^1^ The Feinstein Institutes for Medical Research, Northwell Health, Manhasset, NY, United States; ^2^ Division of Endocrinology, Diabetes & Bone Disease, Icahn School of Medicine at Mt. Sinai, New York, NY, United States; ^3^ Azevan Pharmaceuticals, Bethlehem, PA, United States

**Keywords:** GLP-1 - glucagon-like peptide-1, incretin, GLP-1 agonists, hormone, inflammation, anti-inflammation

## Abstract

Inflammation contributes to many chronic conditions. It is often associated with circulating pro-inflammatory cytokines and immune cells. GLP-1 levels correlate with disease severity. They are often elevated and can serve as markers of inflammation. Previous studies have shown that oxytocin, hCG, ghrelin, alpha-MSH and ACTH have receptor-mediated anti-inflammatory properties that can rescue cells from damage and death. These peptides have been studied well in the past century. In contrast, GLP-1 and its anti-inflammatory properties have been recognized only recently. GLP-1 has been proven to be a useful adjuvant therapy in type-2 diabetes mellitus, metabolic syndrome, and hyperglycemia. It also lowers HbA1C and protects cells of the cardiovascular and nervous systems by reducing inflammation and apoptosis. In this review we have explored the link between GLP-1, inflammation, and sepsis.

## 1 Introduction to GLP-1

Glucagon-like peptide-1 (GLP-1) is a peptide hormone that is produced in the intestine and in multiple other sites that are known for their role in regulating glucose metabolism. GLP-1 is also involved in multiple other physiological processes including appetite, cardiovascular function, and inflammation (1).

Acute Inflammation is central to in-vivo responses to a wide range of challenges including viral and bacteriological infections, and to host repair processes. Chronic inflammation, on the other hand, is associated with conditions like type 2 diabetes, metabolic syndrome, obesity, cancer, arthritis, and bowel diseases like Crohn’s disease and ulcerative colitis (2). Our recent studies have revealed the anti-inflammatory properties of several peptide hormones such as hCG, oxytocin, ghrelin, and vasopressin.(3-6) In this review article, we focus on the anti-inflammatory properties of the incretin hormone ‘Glucagon-like Peptide-1 (GLP-1). Known for promoting glucose homeostasis and weight loss, the anti-inflammatory properties of GLP-1 suggest that it may also blunt inflammation and protect against organ damage (3–6).

## 2 Functions of GLP-1 and its receptors

Glucagon-like peptide-1 (GLP-1) is a 30-31 amino acid long incretin that is produced when proglucagon undergoes post-translational processing. This glucose-lowering agent is secreted by intestinal enteroendocrine L-cells in response to nutritional and inflammatory stimuli and by neurons in the nucleus of the solitary tract in the brainstem. GLP-1 activates a seven transmembrane G protein coupled receptor, GLP-1R. GLP-1 receptors are expressed in pancreatic islet β-cells, pulmonary epithelial cells, atrial cardiac myocytes, vagal afferent neurons, neurons in a number of brain regions, as well as cells lining gastric pits and small intestinal mucosa. The GLP-1R can couple to the Gs or Gq proteins, leading to increases in intracellular cAMP and/or Ca2+ levels and activation of PKA, Epac-2, phospholipase C and ERK1/2 signal transduction pathways. Activation of GLP-1R by GLP-1 or other exogenous agonists, including exendin-4 and liraglutide, decreases inflammatory responses in several animal models like rat heart and whole animal model. The hypoglycemic activity of GLP-1 is associated with the stimulation of glucose-dependent insulin secretion, inhibition of glucagon production and regulation of islet cell proliferation, differentiation, and survival. Under physiological conditions, GLP- 1 is rapidly degraded by dipeptidyl peptidase-4 (DPP- 4) after it is released (7).

## 3 Discovery of GLP-1

**Figure 1:**
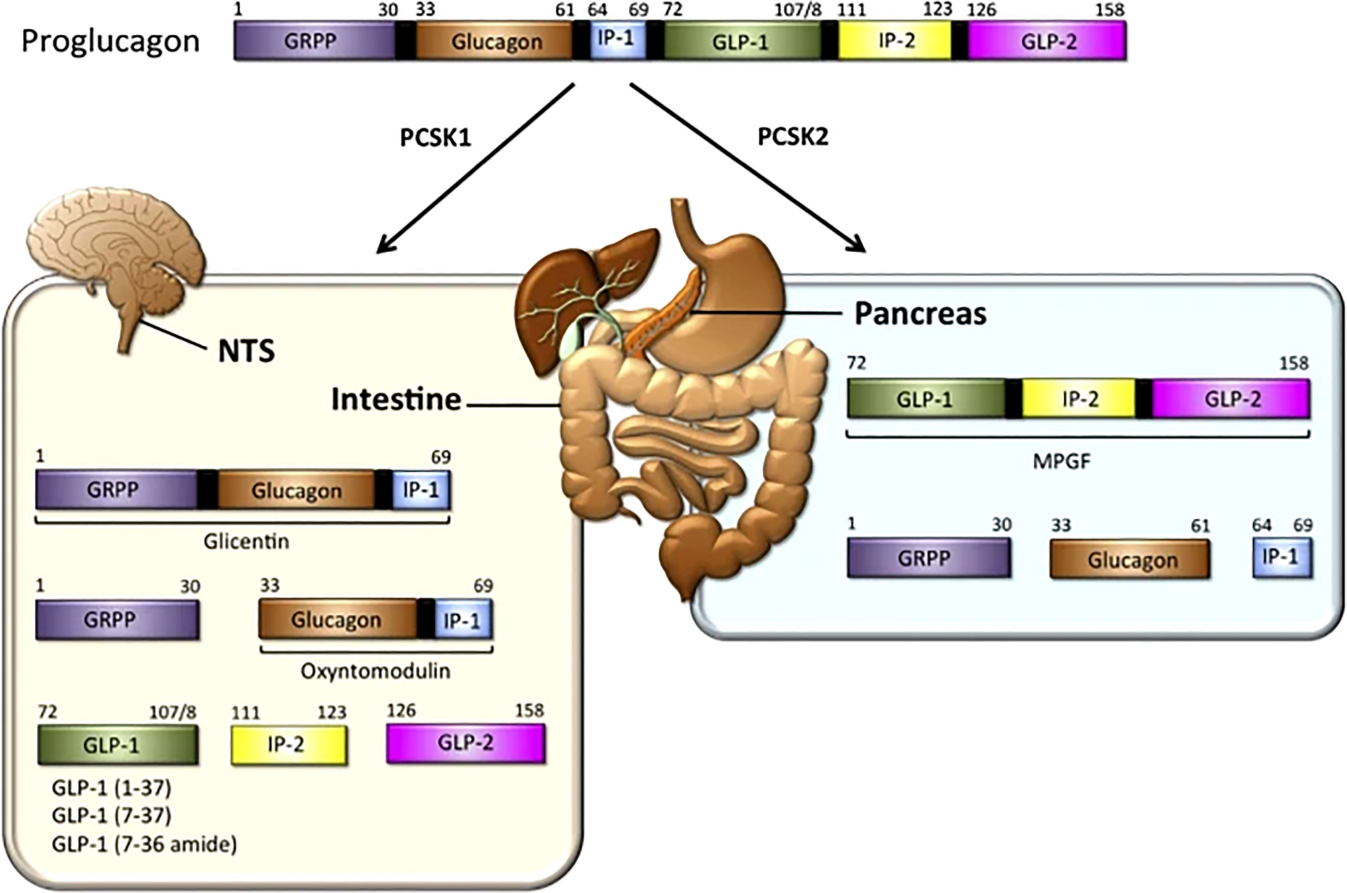
GLP-1 synthesis: In the intestine — The proglucagon precursor gives rise to oxyntomodulin, GLP1 (and its two equipotent, truncated derivatives) and GLP-2. Like the GLPs, the intervening peptides (IP-1 and IP-2) may also have physiological functions (12, 13). In the pancreas, the proglucagon precursor yields glucagon and the glicentin-related pancreatic peptide (GRPP) (12–16) Figure modified from article 12. Figure from Open access (Molecular Metabolism) permissible to re-use under a CC-BY 4.0 license.

In 1923, Charles Kimball and John Murlin, in an attempt to purify commercial insulin, precipitated a pancreatic fraction that had a hyperglycemic effect (8). Identifying it as a secreted factor, they named it ‘Glucagon’ or ‘Glucose Agonist’. In 1959, Roger Unger et al., developed the first antibody that could be used in a radioimmunoassay to detect glucagon in tissue samples and blood (9, 10). In 1966, Ellis Samols, Vincent Marks and others confirmed the presence of glucagon-like immunoreactivity in extra-pancreatic tissue, especially the intestine. Subsequently in 1967, Samols and Marks reported glucagon-like material in pancreatectomized humans, indicating its extra-pancreatic origin (11). In 1968, Roger Unger demonstrated that intraduodenal administration of glucose increased the levels of a circulating glucagon-like substances (9, 10). In contrast to glucagon, the intestinal glucagon-like material stimulated the release of insulin. It was clear that glucagon and the glucagon-like material were distinct entities, and immunohistochemical studies revealed that intestinal cells that were positive for the glucagon-like material had a different morphology from glucagon secreting α-cells. The cells that made glucagon-like material were called L-cells. In 1970 the glucagon precursor, proglucagon, was identified. In the pancreas, proglucagon undergoes post-translational cleavage yielding two fragments. One was a mature glucagon and the other was called the proglucagon fragment. In 1980, the intestinal glucagon-like material, glicentin, was identified along with a smaller species named oxyntomodulin in 1982. Collectively, these studies suggested that proglucagon undergoes tissue-specific processing resulting in formation of glicentin and oxyntomodulin in the intestine, and glucagon plus the N-terminal fragment of glicentin in the pancreas. In the 1980s Joel Habener described a new glucagon-related peptide encoded in the anglerfish preproglucagon cDNA. Subsequently two glucagon-related peptides were identified in rat, bovine, hamster, and human proglucagon. These two peptides are now called glucagon-like peptides 1 and 2 (GLP-1 and GLP-2) as shown in **Figure 1** “The proglucagon precursor (12–16).

## 4 GLP-1 receptor

**Figure 2:**
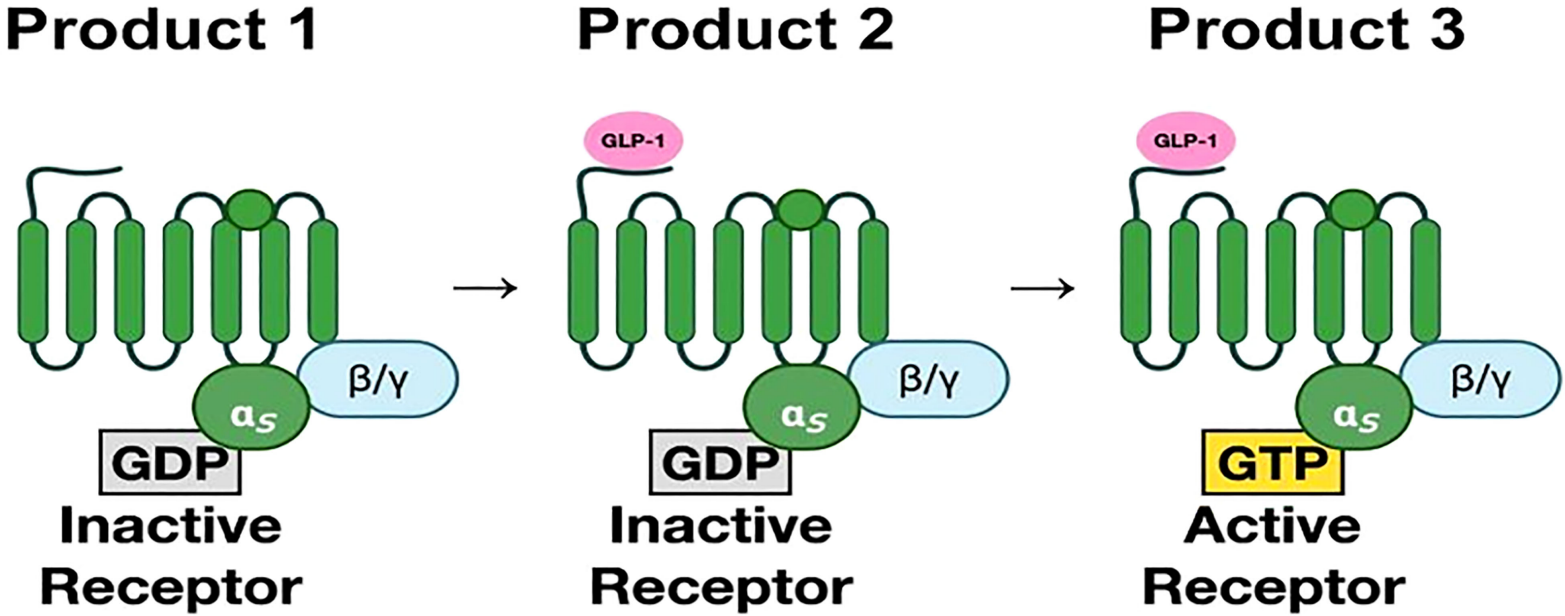
(Original by authors of the manuscript): The seven transmembrane alpha-helices are bound to G-protein subunits (19). These consist of alpha subunit and beta-gamma subunit complexes bound to GDP. In the inactive state, the α-subunit is bound to GDP. Upon binding of GLP-1, GDP is replaced by GTP, which then activates the α-subunit. The α-subunit and GTP complex activate signaling cascades through adenylyl cyclase and phospholipase C (20).The third intracellular loop is most important in receptor signaling.

The GLP-1 receptor is a member of the secretin subfamily (B1) of G-protein coupled receptors (GPCRs). It consists of 463 amino acids (17). These amino acids are arranged in seven transmembrane (7TM) alpha-helices with an N-terminal domain that is located extracellularly and a C-terminal domain that is intracellular. The transmembrane helices are connected by three extracellular and three intracellular loops (17, 18). Ligand binding to its receptor occurs in two stages. The first step involves the binding of the extracellular domain to the C-terminus of the ligand. This causes a conformational shift that leads to attachment of the N-terminus of the ligand to the 7TM domain (18–20). (**Figure 2**) for a more detailed figure, refer to reference 18.

**Figure 3:**
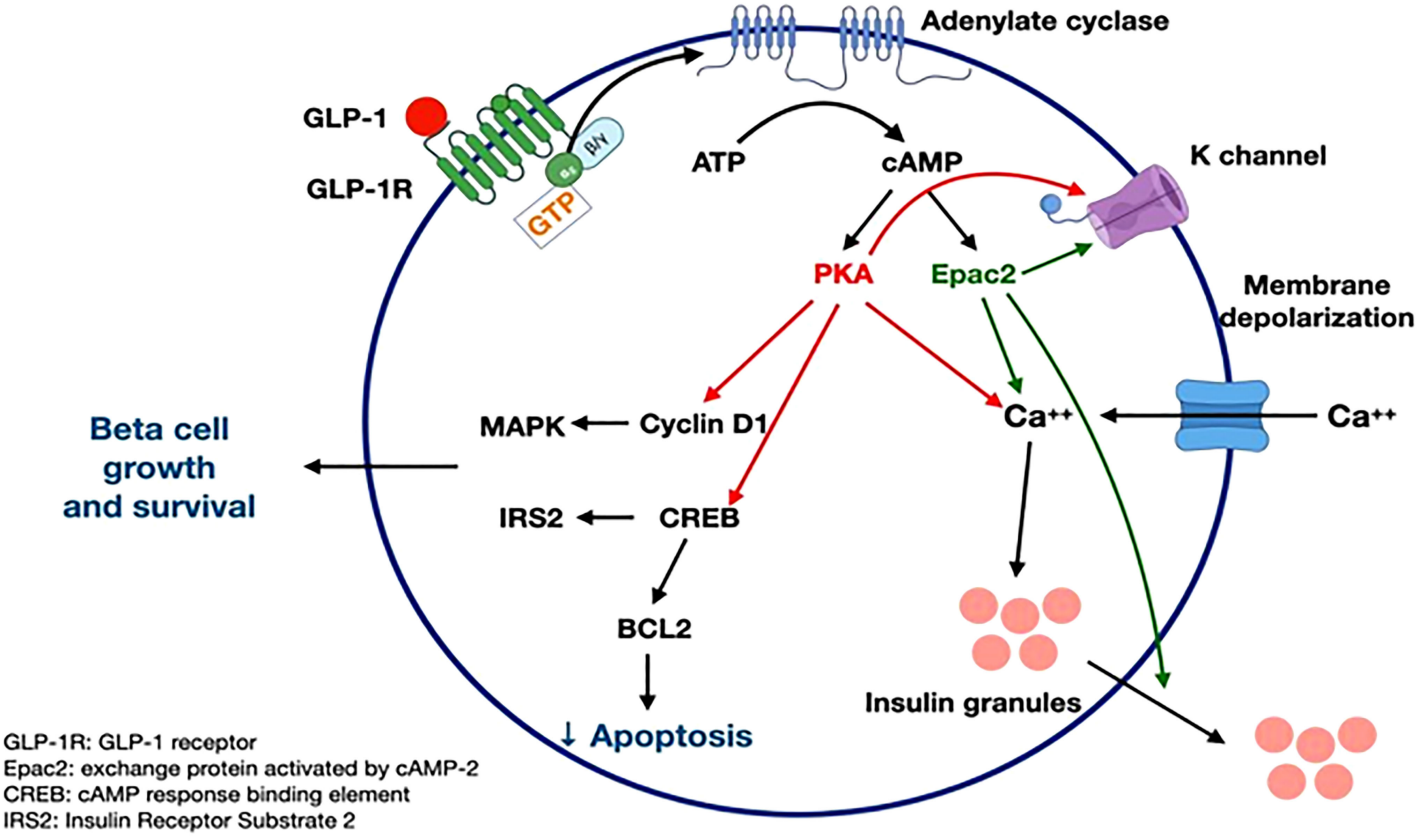
GLP-1 receptor signaling (21-26). Figure modified from article 23. Figure from Open access (Gastroenterology) permissible to re-use under a CC-BY 4.0 license.

GLP-1 receptor signaling occurs primarily through the Gαs stimulatory G protein (21). Coupling of Gαs and Gαq in beta cells of pancreas lead to an increase in cAMP by activation of adenylyl cyclase and phosphoinositol 3 kinase (PI3K) pathway. cAMP activates PKA and Epac-2 signal transduction pathways (22). PKA and Epac-2 inhibit the K-channel, altering Kv currents leading to calcium influx as well as calcium release from the endoplasmic reticulum. This results in calcium-induced release of insulin granules (23). PKA and Epac-2 also activate cyclin D and CREB, leading to beta-cell proliferation, differentiation, and a decrease in endoplasmic reticulum stress response (24). Exenatide decreases ER stress in response to synthetic stressors (25). In mouse models, exendin-4 increases beta cell proliferation by activation of epidermal growth factor receptors (26). Human beta cells exposed to GLP-1 show increased beta cell proliferation (24, 25) (**Figure 3**).

Activation of the GLP-1 pathways decreases the inflammatory response in multiple models. GLP-1 Analog liraglutide improves vascular function in polymicrobial sepsis by reduction of oxidative stress and inflammation (24, 27–31). Exendin in diabetic mice diminishes inflammatory responses by increasing the expression of regulatory T cells (32). Liraglutide has anti-inflammatory effects on endothelial cells by decreasing activation of NF-kB, inhibiting TNF-alpha, and increasing nitric oxide production (28). Like GLP-1 agonists, dipeptidyl peptidase-4 (DPP-4) inhibitors, which block the degradation of GLP-1, also cause attenuation of the inflammatory responses. Sitagliptin decreases the LPS-inflammatory response by inhibiting the NF-kB pathway. This leads to decreased production of proinflammatory cytokines including TNF-α, IL-6, IL-1β and decreased expression of COX-2 in cardiomyocytes (33).

## 5 GLP-1 and various organs

**Figure 4:**
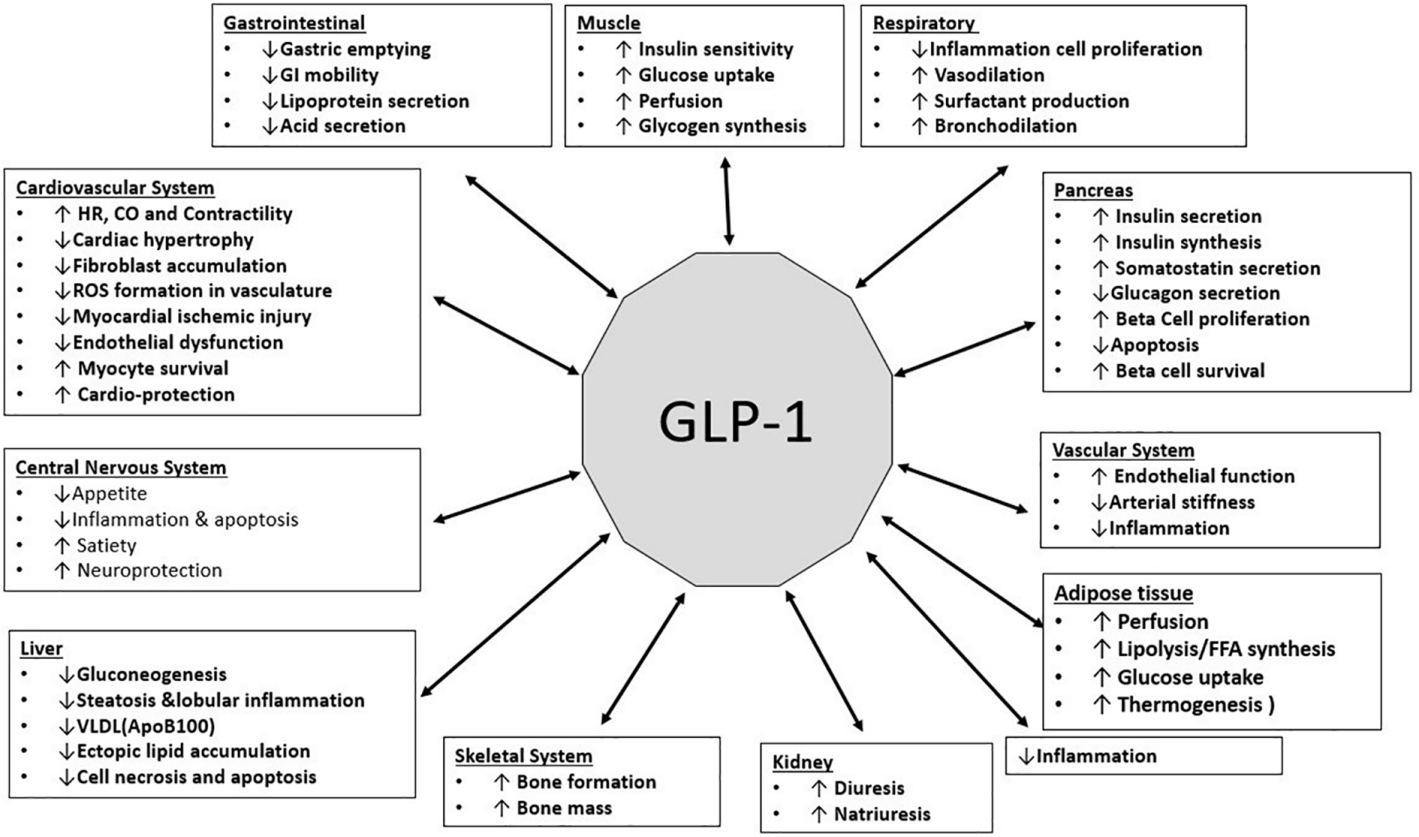
(Original by authors of the manuscript): Effects of GLP-1 on various organ systems (34–39).

GLP-1 has been shown to carry out numerous protective and regulatory functions in different organ systems. The functions are illustrated in the Figure (**Figure 4**).

## 6 GLP-1: anti-inflammatory effects

### 6.1 Cardiovascular system

The antioxidant and anti-inflammatory effects of GLP-1 protect the cardiovascular system. GLP-1 levels are elevated in patients post myocardial infarction. Administration of GLP-1 analogs (or DPP-4 inhibitors, which inhibit the degradation of GLP-1), decreased cardiovascular and thrombotic complications in animal models of LPS-induced sepsis. They also suppressed inflammation and formation of reactive oxygen species (ROS) in vasculature, resulting in vasorelaxation and amelioration of hypotension. Moreover, reduced organ damage by thrombotic occlusion in the lung has been reported in LPS-induced sepsis due to improvement in microvascular circulation by GLP-1 analogs. In a polymicrobial model of sepsis induced by cecal ligation and puncture, a GLP-1 analog ameliorated vascular inflammation and oxidative stress by improving endothelial function (28)

In a cardiac fibrosis model, the GLP-1 analogue liraglutide reduced vascular reactivity, cardiac hypertrophy, fibroblast accumulation, collagen deposition and MCP-1 production (40). Another GLP-1 analogue, exendin-4, also prevented cardiac remodeling and diastolic dysfunction in an experimental diabetes model. This was associated with a reduction in macrophage infiltration, lower expression of IL-1β and IL-6, and an increase in IL-10 in the heart (41).

GLP-1 improved left ventricular function in patients with chronic heart failure and in dogs with dilated cardiomyopathy. Survival rates after myocardial infarction also improved after GLP-1 administration. Sitagliptin, a DPP-4 inhibitor, improves myocardial response in coronary artery disease patients. LPS-induced cardiac dysfunction recovered in DPP-4 deficient rats after treatment with sitagliptin. Exendin-4 and DPP-4 deficiency prevented vasoconstriction and multiple organ injury after LPS treatment, and improved survival in endotoxemic rats (31).

In animal studies, GLP-1 and its analogs reduced macrophage infiltration in blood vessels, and production of pro-inflammatory cytokines such as IL-6, IL-1β, TNF-α, and CRP. It has been speculated that liraglutide, a GLP-1 analog, suppresses cytokine release in bacterial septic shock and in SARS-CoV-2 viral sepsis. GLP-1 analogs and DPP-4 inhibitors have shown promise in animal models of cardiovascular disease. Studies in humans should be done (42, 43) (**Table 1**).

**Table 1:** Efficacy of GLP-1 agonists efficacy (adapted from UpToDate) (44).

	Elimination half-life	Glycemic efficacy (reduction in A1C in % points)*	Cardiovascular outcomes ASCVD/HF	Nephropathy^¶^	Retinopathy^Δ^	Cardiovascular Overall mortality
Long-acting GLP-1 receptor agonists (more pronounced effect on fasting glucose)						
Dulaglutide	5 days	–1 to –1.5	Benefit	Benefit	Neutral	Benefit
Efpeglenatide	6 to 7 days	–1 to –1.11	Benefit	Benefit	?	?
Exenatide	8 to 14 days	–1.5 to –1.6	Neutral	?	Neutral	Benefit
Liraglutide	11 to 15 hrs	–0.8 to –1.5	Benefit	Benefit	Neutral	Benefit
Semaglutide	6 to 7 days	–1.5 to –2	Benefit	Benefit	Unexpected increase in retinopathy outcomes^◊^	Benefit
Short-acting GLP-1 receptor agonists (more pronounced effect on postprandial glucose)						
Exenatide	2 to 3 hrs	–1	?	?	?	?
Lixisenatide	3 to 5 hrs	–0.8 to –1	Neutral	Neutral	?	Benefit
Dual-acting GLP-1 and GIP receptor agonists						
Tirzepatide	5 days	–2 to –2.5	?^§^	?	?	?

GLP-1, glucagon-like peptide 1; A1C, glycated hemoglobin; ASCVD, atherosclerotic cardiovascular disease; HF, heart failure; SubQ, subcutaneously; ?, inadequate data; GIP, glucose-dependent insulinotropic polypeptide; eGFR, estimated glomerular filtration rate.

*Reduction in A1C is dependent upon a number of factors, such as baseline A1C and background therapy. In trials directly comparing shorter- versus longer-acting GLP-1 receptor agonists, longer-acting had better glycemic efficacy.

¶Nephropathy is defined as elevated albuminuria, reduced eGFR (usually <60 mL/min/1.73 m2), or both.

ΔRetinopathy outcomes were not systematically evaluated or adjudicated.

◊The higher rate of retinopathy complications with subcutaneous semaglutide was unexpected and may be a consequence of rapid glycemic control similar to that seen in other settings. If subcutaneous semaglutide is prescribed to a patient with diabetic retinopathy, titrate slowly to avoid rapid declines in A1C and perform retinal screening within 6 months of drug initiation to detect progression of retinopathy.

§In preliminary trials, tirzepatide did not increase the risk of major cardiovascular events.

### 6.2 Gastrointestinal system

GLP-1 is secreted into the distal intestine by enteroendocrine L cells in response to nutrient ingestion (42). GLP-1 receptors are widely distributed in the gastrointestinal tract, pancreas, heart, lungs, kidneys, and nervous system. These receptors contribute to the wide range of physiological functions (45). Besides metabolic effects, GLP-1 improves mucosal integrity and diminishes inflammation (42, 46). Exendin-4, a GLP-1 mimetic peptide, decreases the production of pro-inflammatory cytokines, and diminishes the enteric immune response. GLP-1 decreases production of pro-inflammatory cytokines, mainly by downregulating NF-κB phosphorylation and nuclear translocation (45).

Several recent studies have suggested that GLP-1 should be considered as a treatment for a wide range of intestinal diseases, including Inflammatory bowel diseases, intestinal mucositis, coeliac disease and short bowel syndrome (45). GLPs, (including GLP-1, GLP-2 and DPP-4) have recently gained increased attention from researchers studying Inflammatory bowel diseases (IBDs).

IBDs including Crohn’s disease and ulcerative colitis are chronic relapsing-remitting diseases with multifactorial etiologies and complex pathogenesis. The Incidence and prevalence of IBDs are rising globally. GLPs including GLP-1 regulate weight and glycemia. GLP-1 also inhibits gastric emptying, decreases food ingestion, and increases crypt cell proliferation. It also improves intestinal growth and nutrient absorption. GLPs have been proposed to improve tissue healing of injured epithelium, regulate T-cell growth and function, control innate immune cells such as macrophages and dendritic cells, and lower pro-inflammatory cytokines in IBD (47) (**Table 2**).

**Table 2:** GLP-1 analogues under investigation *in vitro* and *in vivo* (animal and human studies).

Models	Treatments	Results	References
In-vitro study Macrophage RAW 264.7 cell culture	Pre-treated with exendin-4 for 6hrs followed by LPS for 24hrs	Exendin-4 inhibits production of many LPS-induced inflammatory factors, thereby decreasing production of ROS reactive oxygen species.	(Lu et al., 2019) (27)
In-vivo animal studies DSS-induced colitis in mouse model	GLP-1 self-associated with PEGylated phospholipid micelles i.p	GLP-1-SSM (sterically stabilized phospholipid micelles) improve architecture of the intestine, partially preserve goblet cell number, decrease IL1-β secretion and improve diarrhea induced by DSS.	(Anbazhagan et al., 2017) (48)
DSS-induced colitis in mouse model — Ischemia/reperfusion	—	I.P or I.V administration of LPS caused a significant rise in plasma levels of GLP-1 through the TLR-4 mechanism.	(Lebrun et al., 2017) (46)
DSS-induced colitis in mouse with GLP-1R knockout	Exendin-4 (GLP-1 agonist) s.c.	DSS-induced colitis in GLP-1 R knockout mice showed dysregulation of intestinal gene expression, as well as abnormal representation of microbes in feces and increased sensitivity to intestinal injury. Also, Exendin-4 administration caused significantly increased expression of genes encoding cytokines and chemokines in gut injury.	(Yusta et al., 2015) (49)
Colonic smooth muscle cells of male BALB/c mice cultured in DMEM	LPS+/-Exendin-4	Exendin-4 Inhibited production of pro-inflammatory cytokines including TNF-α and IL-1 α in LPS-induced inflammation in mouse model.	(Al-Dwairi et al., 2018) (50)
Wistar rat model	a) GLP-1 injected i.c.v b) GLP-1 receptor antagonist, Exendin 9-39 I.c.v and i.p	Centrally injected Exendin 9-39 inhibited the gastroprotective effects of GLP-1 agonists, suggesting that this effect is managed by central mechanisms.	(Işbil Büyükcoşkun et al., 2007) (51)
MPTP-treated Parkinson Disease mouse model Human A53T α-synuclein transgenic PD mouse model (MPTP=1-methyl-4-phenyl-1,2,3,6-tetrahydropyridine)	CCK analogues or Liraglutide and GLP-1 analogues i.p	CCK analogues or GLP-1 analogues restored the disruption of intestinal tight junction, reduced colonic inflammation, inhibited colonic dopaminergic neuron reduction and the accumulation of α-synuclein oligomers in the colon of both PD mouse models.	(Su et al., 2022) (52)
Human study a) Healthy volunteers b)Ischemia/reperfusion injury model of human gut	—	a) 3hrs after LPS injection plasma GLP-1 levels rose significantly. b) 45 min after ischemia in the human intestine, GLP-1 levels rose significantly and returned to baseline after reperfusion.	(Lebrun et al., 2017) (46)

### 6.3 Hepatobiliary system

GLP-1 based therapies have shown promise in liver diseases e.g. non-alcoholic fatty liver disease (NAFLD) and non-alcoholic steatohepatitis (NASH). In recent years, the prevalence of non-alcoholic fatty liver disease (NAFLD) has continued to rise, and 10%-25% of NAFLD cases progress to non-alcoholic steatohepatitis (NASH). 10%-15% of NASH cases will develop into hepatocellular carcinoma, approximately 700,000 people die from the disease each year (53).

Nonalcoholic steatohepatitis is associated with inflammation of the liver, driven by an aberrant accumulation of fat. In rats fed with a high-fat diet, treatment with liraglutide, a GLP-1R analog, reduced steatosis and lobular inflammation compared to the saline-injected group. Exendin-4, a GLP-1R agonist, was shown in another study to lower hepatic production of the inflammatory markers TNF-, IL-1, and IL-6, as well as macrophage markers cluster of differentiation 68 (CD68), and F4/80 in mice fed a western-type (high fat) diet (54).

C-reactive-protein (CRP) is produced by the liver and is a marker of inflammation. Liraglutide produced a significant decrease in the mean concentration of CRP in a retrospective investigation of 110 obese patients with type 2 diabetes mellitus, indicating its potential as an anti-inflammatory drug. Exenatide plus metformin caused a significant reduction in baseline CRP and TNF-α. These findings show that GLP-1-based treatments improve fatty liver disease in rats and humans *via* reducing inflammation (42).

NAFLD is associated with cell death and fibrosis that ultimately progress to cirrhosis. In obese patients with NAFLD, Fibroblast growth factor-21 protein (FGF21) and RNA levels are higher in the liver. Treatment with GLP-1R agonists reduced the level of FGF21. This supports its use in cirrhosis. Note that 80% of patients who develop hepatocellular carcinoma had cirrhosis beforehand (55, 56).

**Figure 5:**
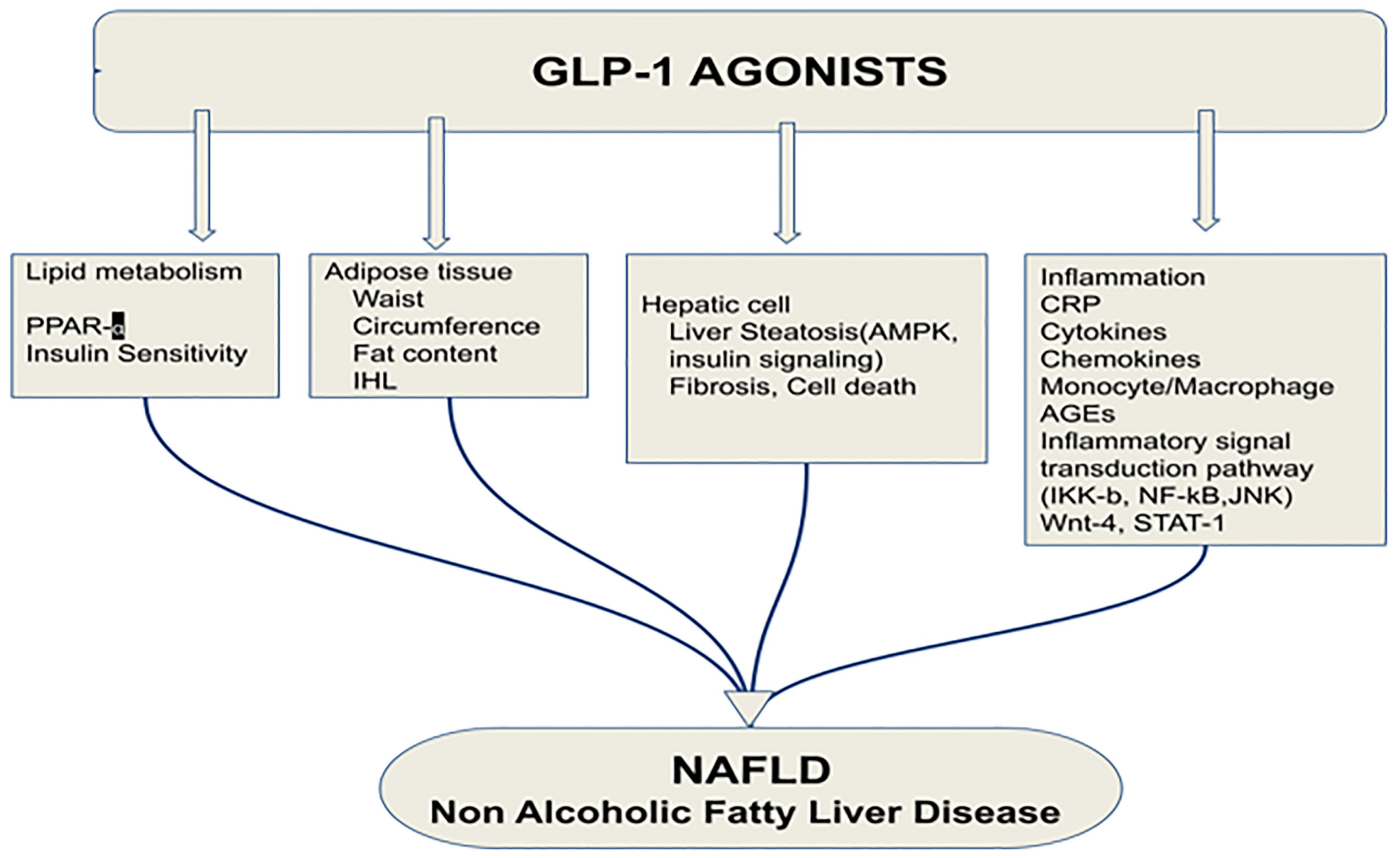
Effects of glucagon-like peptide-1 receptor agonist on non-alcoholic fatty liver disease. PPAR-α, Peroxisome proliferator-activated receptor; IHL, intrahepatic lipids; AMPK, AMP-activated protein kinase; CRP, C reactive protein; AGEs, Advanced glycation and end products; JNK, c-Jun NH2-terminal kinase; GLP-1RA, Glucagon-like peptide-1 receptor agonist; NAFLD, Non-alcoholic fatty liver disease (53). Modified figure from reference 53. Figure from Open access (World Journal of Gastroenterology) permissible to re-use under a CC-BY 4.0 license).

GLP-1RA significantly reduced cell necrosis and apoptosis, the two major forms of liver cell death. Hepatic cell death mainly includes two forms: apoptosis and cell necrosis. Gupta et al. showed that a GLP-1RA significantly reduced cell necrosis and apoptosis.The reduction of abdominal visceral adiposity by GLP-1RAs results in a reduction in liver fat content that can alleviate NAFLD. The ability of GLP-1 to reduce fat is due to its binding to a specific GLP-1R present in adipose tissue (57). Vendrell et al. confirmed the expression of GLP-1R in mature adipose cells by the detection of the mRNA and protein (58). A 6-month-long treatment with GLP-1RAs in obese patients with T2DM resulted in significant reductions in intrahepatic lipids (IHL). In addition, the median relative reduction in IHL was 42% (53) (**Figure 5**).

### 6.4 Central nervous system

Glucagon-like peptide-1 is produced in the brainstem and has numerous functions, including neuroprotection (59–61) GLP-1 and GLP-1 analogs can cross the blood-brain barrier (62–68) GLP-1 receptors have been observed in the neurons of the nucleus tractus solitarius that project to GLP-1R–expressing regions in the hindbrain, hypothalamus, including the paraventricular nucleus (PVN), dorsal medial nucleus of the hypothalamus, and arcuate nucleus (ARC) (42, 64, 66, 67, 69, 70). GLP-1-based therapies have anti-inflammatory effects on multiple tissues (42, 53, 71–73).

Chronic inflammation is a significant risk factor for many neurodegenerative disorders, e.g., Alzheimer’s disease and Parkinson’s disease (42, 74–78).

#### 6.4.1 Parkinson’s disease 

The prevalence of Parkinson’s disease has been rising in recent years (79, 80). It is the second most common chronic neurodegenerative disease and affects between 1% - 2% of people above age 60 and 4% of those above age 80 (81–87). Parkinson’s disease occurs when dopaminergic neurons in the substantia nigra pars compacta form Lewy bodies and gradually die (88–90). The Lewy body is an abnormal aggregate containing alpha-synuclein. Most Parkinson’s disease treatments focus on managing symptoms by replacing dopamine and improving dopaminergic signaling, but these treatments fail to address the underlying cellular degeneration (64, 91). Since dopamine breaks down to form reactive oxygen species, it may contribute to disease progression (92, 93). Activation of microglia plays a crucial role in spontaneous Parkinson’s Disease in humans (64, 94–96). MPTP (1-methyl-4-phenyl-1,2,3,6-tetrahydropyridine) induces Parkinson’s disease in rodents. MPTP is a pro-drug for the neurotoxin MPP+ (1-methyl-4-phenylpyridinium). This agent destroys dopaminergic neurons in the substantia nigra (81, 97–104).Exendin- 4, a GLP-1 R agonist, has inhibitory effects on microglial activation and greatly reduces the expression of TNF-α and IL-1β caused by MPTP (67, 105–107). Exendin-4 inhibits 6-hydroxydopamine (6-OHDA)-induced dopaminergic cell death in neuronal culture. The intraventricular administration of GLP-1 protects mice from MPTP-induced dopaminergic cell loss (64, 86, 108, 109).

#### 6.4.2 Alzheimer’s disease

Alzheimer’s disease is the most common form of dementia; it is responsible for 60–70% of cases (110, 111). About 1 person in 9 (10.8%) in the US population age 65 and older has AD (112). People 65+ years of age in Europe had a pooled incidence rate of AD of 19.4 per 1000 person-years (113–115). The Alzheimer’s disease population increased by 5% from age 65 to 73, 13.1% from age 75 to 84, and 32% from age 85 and older (112, 116). Alzheimer’s disease was the seventh-leading cause of death in 2020 and 2021 (112).

**Figure 6:**
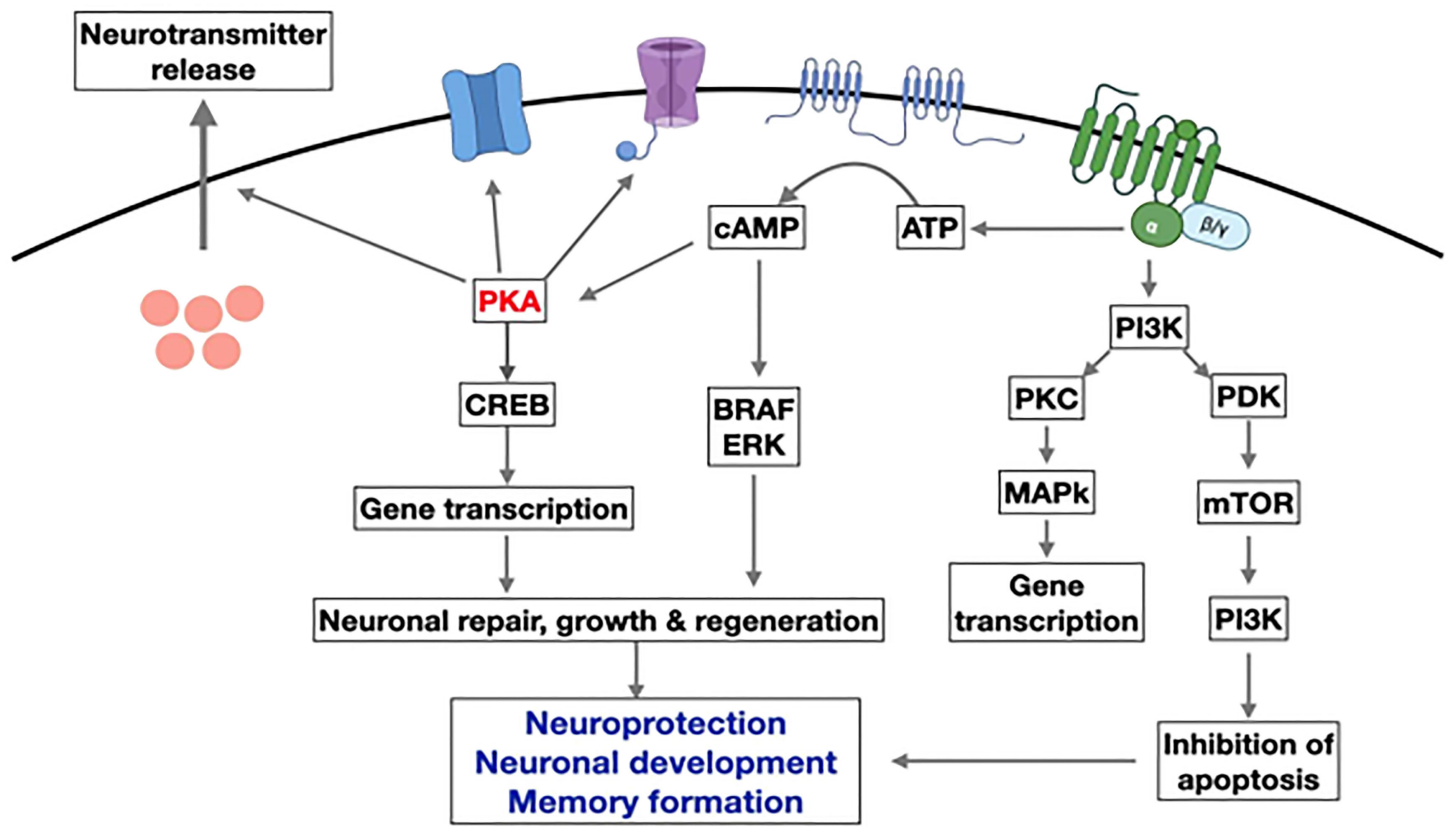
Overview of the main pathways induced by GLP-1 in neurons. Activation of the GLP-1R activates adenylyl cyclase and increases cAMP levels. This activates PKA and other downstream kinases related to growth factor signaling. GLP-1 supports neurogenesis, reduces inflammation, and inhibits apoptosis while improving learning and memory in the hippocampus.(modify from reference 130) (AATP, adenosine triphosphate; cAMP, Cyclic adenosine monophosphate; CREB, cAMP response element binding protein; PKA, protein kinase A; PI3K, phosphatidylinositol-3 kinase; PKC, protein kinase c; mTOR, Mammalian target of rapamycin; ERK, extracellular signal-regulated kinase; BRAF, v-raf murine sarcoma viral oncogene homolog B1.) Figure modified from reference 130, Open access (Peptides journal) permissible to re-use under a CC-BY 4.0 license).

In AD, IL-1 beta is significantly increased in the frontal cortex and hippocampus and may contribute to cognitive dysfunction by promoting the synthesis of amyloid precursor protein (117, 118). GLP-1 therapies may have preventive and restorative effects on Alzheimer’s disease (42, 119). Exogenous GLP-1 (7–36) amide administration inhibited IL-1 beta transcription and prevented beta-induced amnesia and cell death (36, 59, 113, 114). Also, it restores learning and memory by stimulating LTP (long-term potentiation) (60, 120–122). In a rodent model, neuroinflammation was reduced due to suppression of TNF-alpha when GLP-1 exenatide (20 ug/kg/day) was given intraperitoneally. The peptide improved memory and prevented the loss of hippocampal neurons (111, 123). Treatment with liraglutide in a mouse model of Alzheimer’s disease reduced the inflammatory response in the cortex by decreasing the number of activated microglia (60, 65, 107, 124, 125). Mice that express two human mutant genes linked to early-onset Alzheimer’s disease develop a chronic inflammatory response (126). In these animals, D-Ala2-GIP reduces the activation of microglia and astrocytes in the brain, decreasing the release of pro-inflammatory cytokines and oxidative stress (127).Microglia and astroglia express GIP receptors (128, 129). Activating them reduces central inflammatory responses. GIP receptor activation increases microglia expression of key growth factors such as brain-derived neurotrophic factor(BDNF), glial cell-line derived neurotrophic factor (GDNF), and nerve growth factor (NGF) in a phosphoinositide 3-kinase (PI3K) and protein kinase A (PKA) dependent manner (130) (**Figure 6**).

Brain irradiation has been demonstrated to increase the expression of IL-6, IL-1β, and IL-12p70 cytokines. Liraglutide reduces the proinflammatory cytokine gene expression caused by X-ray irradiation (42, 131).

**Figure 7:**
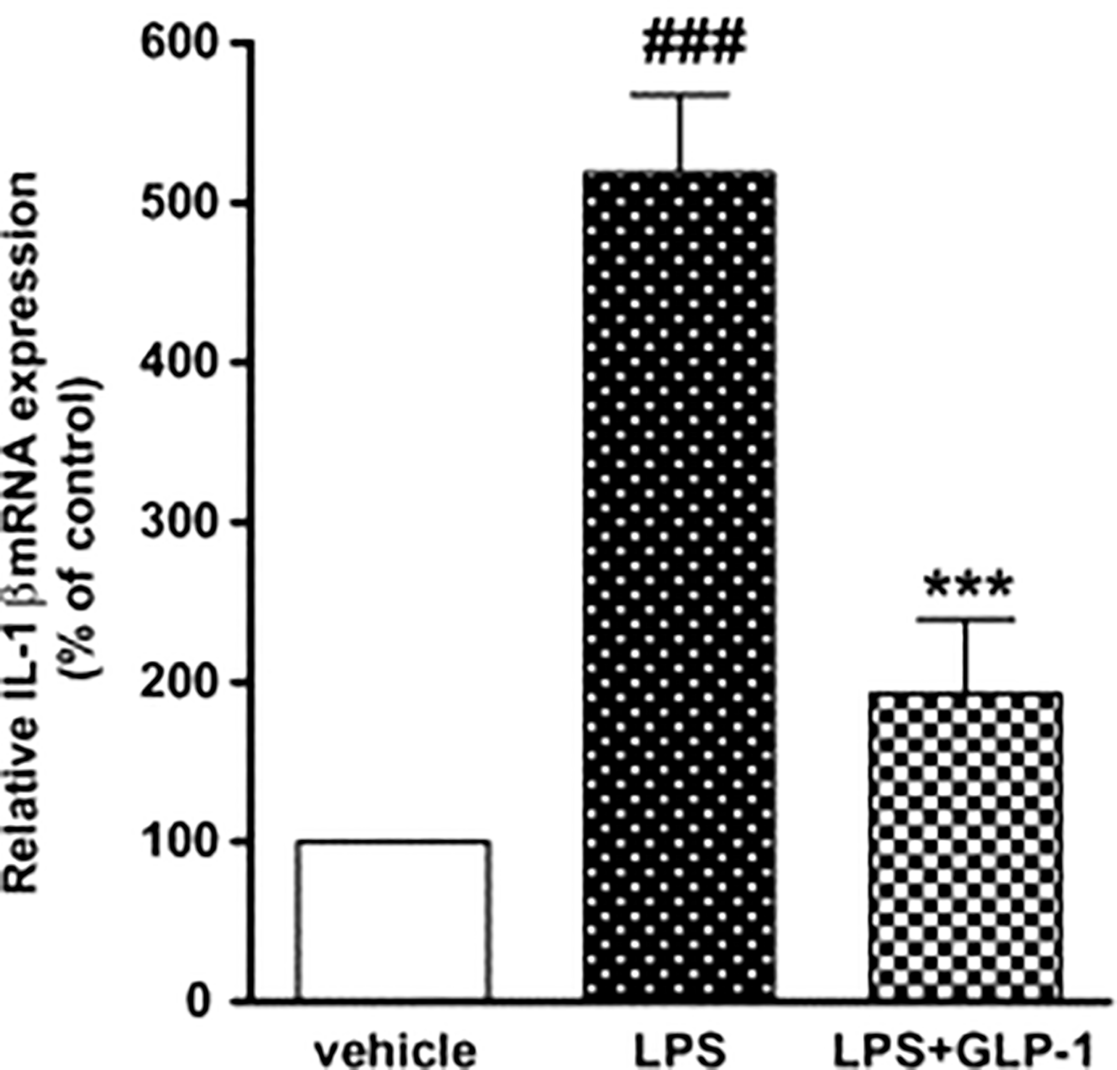
Impact of GLP-1 on LPS-induced IL-1b mRNA production in rat astrocytes. ELISA investigation was performed at 360 min after LPS (100 ng/mL) or vehicle treatment with or without GLP-1 (1 mM) (72). Data represent the mean ± SEM. ***p<0.001. Figure modified from Open access (Neuroscience Research Journal) permissible to re-use under a CC-BY 4.0 license). ###p<.001.

In a study on rats, when cultured astrocytes were stimulated by LPS, IL-1b mRNA expression increased temporally. GLP-1 therapy decreased IL-1b mRNA production compared to the LPS alone-treated cultures (67, 72). The GLP-1 suppresses TNF-alpha and associated cytokines in microglia (**Figure 7**).

### 6.5 Stroke models

Strokes in the elderly can cause permanent neurological damage and are among the leading causes of death. Patients who have hyperglycemia and diabetes mellitus type 2 (T2DM) have a higher stroke frequency than those who do not have these conditions (132, 133). Stimulating GLP-1Rs with exendin-4 reduces brain damage and improves stroke outcomes (108, 132, 134, 135). Exendin-4 suppresses oxidative stress, inducible nitric oxide synthase (iNOS) expression, and cellular apoptosis after ischemia/reperfusion injury (135, 136).

**Figure 8:**
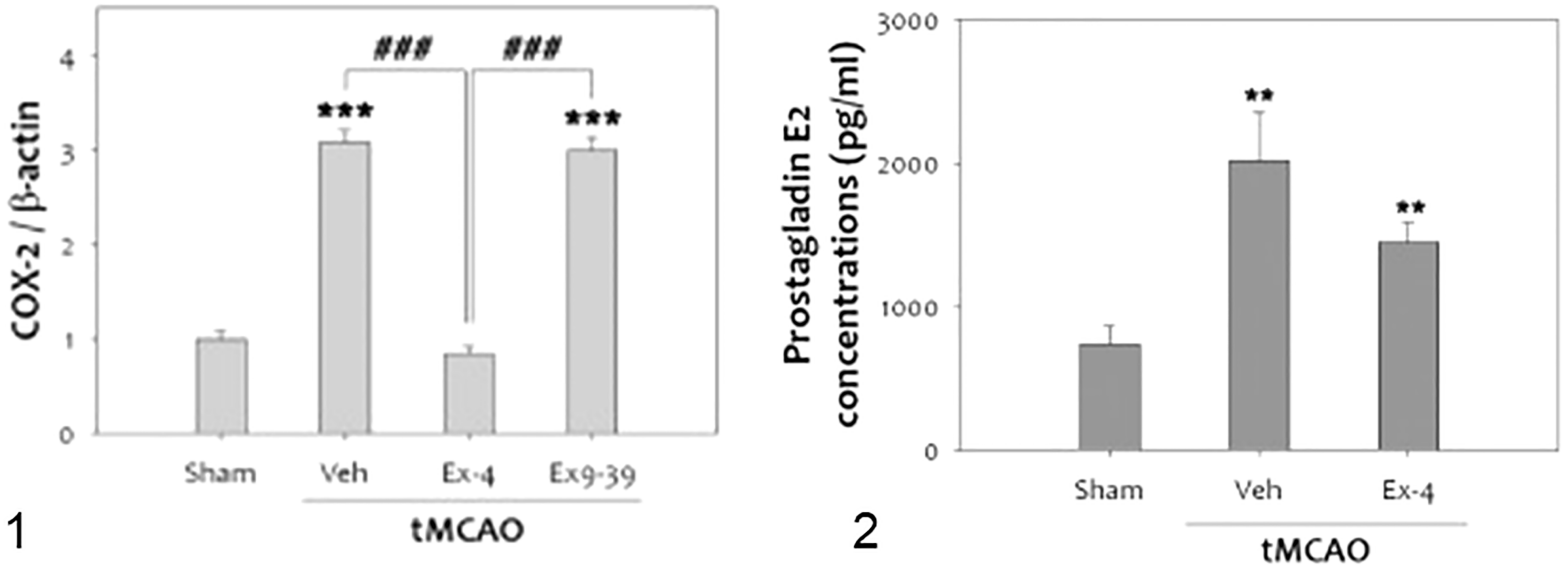
tMCAO, Transient middle cerebral artery occlusion (132): 1-The level of COX-2 was significantly increased at 48 h after tMCAO. Treatment with ex-4 restored COX-2 to the basal level after tMCAO in the rat brain. Ex9-39 treatment increased COX-2 levels as much as vehicle Group.(***p<0.001, compared to sham operated group, ###p<0.001, compared to chemical group) 2-The level of PGE2, which is product of COX-2 activity, was increased by 1 h tMCAO, but this level was attenuated by ex-4 (n =5, **p<0.01, compared to the sham-operated group. (1) Data represent the mean ± SEM. ***p<0.001, ^###^p<0.001, (2) Data represent the mean ± SEM. **p<0.01. Figure modified from Open access (Experimental Neurobiology) permissible to re-use under a CC-BY 4.0 license).

It is well known that inflammation contributes to the progression of brain damage following ischemia/reperfusion injury, and that COX-2 is a significant mediator of oxidative damage (132, 137). Activation of GLP-1Rs has anti-inflammatory effects in cerebral ischemia. COX-2 expression in rats was reduced when they were treated with exendin 9-39 (antagonist) after ischemia was induced (132) (**Figure 8**).

**Figure 9:**
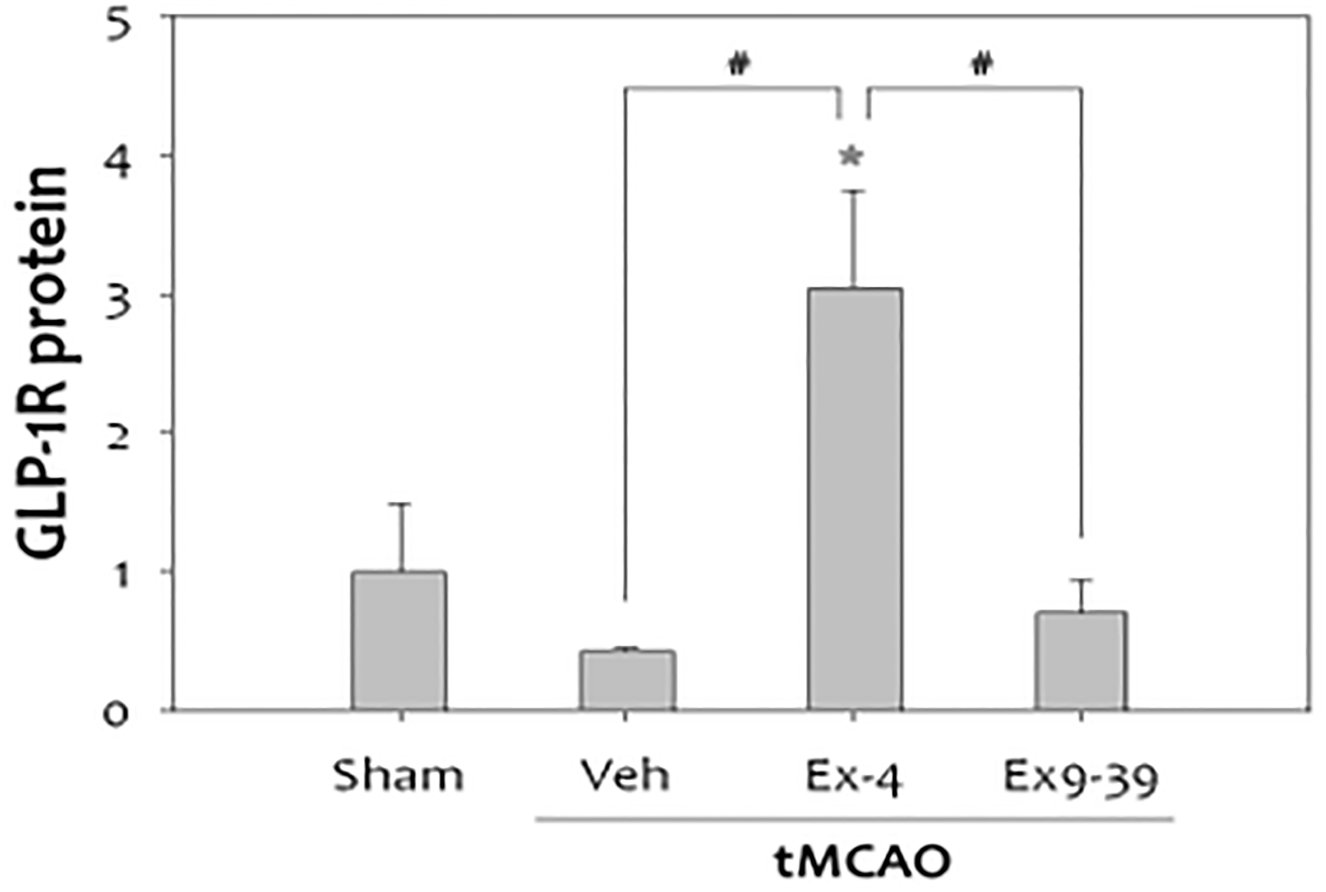
Treatment with exendin-4 was accompanied by increased expression of GLP-1R, while treatment with the GLP-1R antagonist, exendin-9-39 (antagonist), did not show this neuroprotective effect in stroke(n=4, *p<0.05, compared to sham-operated group, #p<0.01, compared to the chemical-treated group). Data represent the mean ± SEM. *P<.05; #P<0.01 (132). Figure modified from Open access (Experimental Neurobiology) permissible to re-use under a CC-BY 4.0 license).

There was a reduction in GLP-1R expression in rat brains after cerebral ischemia. Furthermore, administration of the GLP-1R agonist exendin-4 *in vivo* and *in vitro* proved protective (132, 138, 139) (**Figure 9**).

GLP-1R elevates cAMP levels and activates protein kinase A (PKA) signaling. Adding GLP-1 to neurons increases cAMP, which indicates receptor activation (108, 132, 140). In mice with transient focal cerebral ischemia, exendin-4 treatment increased cAMP and activated the cAMP response element-binding protein (CREB) compared with vehicle-treated mice (135).

### 6.6 Respiratory system

GLP-1 plays an important role in respiratory system homeostasis (141). Glucagon-like peptide-1 receptors (GLP1-R) are found in airway structures as well as vascular and smooth muscle tissues (142). Covid victims who took GLP-1R agonists had fewer hospital admissions (143).

## 7 GLP-1 in obstructive lung disease and asthma

Asthma affects about 25 million people in the US and more than 330 million people world-wide (144). GLP-1 receptor agonists decreased allergic responses in asthma by preventing the activation of NF-kB leading to decreased release of proinflammatory cytokines (IL-5, IL-13, IL-33) and neutrophils, eosinophils, basophils and CD4+ T cell numbers (142, 145). Exendin-4 also relaxes bronchial smooth muscles by acting on the cAMP-PKA pathway (144).

A recent study demonstrated that GLP-1 agonists improve survival and lung function in mouse models of asthma and COPD. The results showed that GLP-1R agonists have therapeutic potential in the treatment of chronic obstructive pulmonary diseases by decreasing the severity of acute exacerbations. The anti-inflammatory effects of GLP-1 agonists in obstructive disease was evident in studies of female C57BL/6 mice. There was a decrease in CD31+ endothelial cells in lung tissues after agonist treatment (146). Trials in humans have also shown that liraglutide administration improves forced vital capacity (147).

GLP-1 causes an increase in cAMP concentration and phosphorylation of endothelial nitric oxide synthase (NOS). Nitric oxide produced as a result may be responsible for the effects of GLP-1 on vasodilation, surfactant production and bronchodilation.

GLP-1 also activates protein kinase A (PKA), which inhibits pro-inflammatory mediators such as nuclear factor kappa light chain enhancer of activated B cells (NF-kB), receptor of advanced glycation end products (RAGE) and asymmetric dimethylarginine (ADMA), an endogenous NOS inhibitor. These mediators play a central role in obesity-related asthma by increasing inflammatory cell proliferation and infiltration, airway remodeling, airway hyperreactivity and bronchoconstriction (148). A recent study showed that bronchodilation caused by GLP-1 analog Exendin-4 was inhibited by GLP-1 receptor blockers. or cAMP-PKA antagonists. Dipeptidyl peptidase-4 (DPP-4), which degrades GLP-1, is expressed in the lungs. Allergens cause upregulation of DPP-4 expression. DPP-4 activates pro-inflammatory pathways (MAPK and NF-kB) and also increases reactive oxygen species, AGE and RAGE gene expression (148).

## 8 GLP-1 in acute lung injuries

Acute lung injury is one of the most serious complications of sepsis. LPS administration in mice leads to endotoxemia and sepsis. Inflammation in sepsis can wash out surfactant leading to the development of acute respiratory distress syndrome (ARDS). GLP-1 agonists have a protective effect in acute lung injury and ARDS. GLP-1 promotes the production of surfactant through PKA-dependent and PKC-dependent mechanisms (149, 150). Following LPS injections in mice, co-administration of GLP-1 diminishes the decline in surfactant levels (142).

## 9 Studies with liraglutide

Liraglutide has benefits in the treatment of acute lung injury. It increases surfactant protein A (SPA) expression in type 2 pneumocytes (151). Pre-administration of liraglutide in mice with LPS-induced acute lung injury decreases the concentration of neutrophils and pro-inflammatory cytokines (IL-1B & IL-18) in the bronchoalveolar lavage fluid by down-regulating the expression of NLRP3 inflammasome (152). Liraglutide also reduced the levels of TNF-α, IL-1β, IL-6 and the severity of lung injury in mouse models (153). Use of liraglutide along with mesenchymal stem cells (MSCs) for treatment of acute lung injury inhibits MSC apoptosis *via* PKA/β-catenin pathway and improves their efficacy (141).

## 10 Studies with exendin-4

Decreased FOXA2 expression leads to increased mucus secretion in the lungs of asthma, COPD, and bronchiectasis patients. Exendin-4, (GLP-1R agonist), increases FOXA2 expression and restores mucus homeostasis in Pseudomonas aeruginosa infected lungs. It decreases mucin expression by pyocyanin (154). It protects against hyperglycemia-induced lung injury by reducing oxidative injury and glucose levels and stimulating the proliferation of pneumocytes. On the other hand, Oztay et al. reported that exendin-4 administration led to increased lung injury by increasing collagen accumulation around pulmonary vessels (155).

## 11 GLP-1 in lung fibrosis

Along with its beneficial effects in obstructive diseases and acute lung injury, GLP-1 also protects against lung fibrosis. In mice exposed to bleomycin, significant reductions in inflammation and fibrosis were seen after GLP-1 therapy due to reduction in NF-kB signaling and TGF-β1 levels (156).

## 12 GLP-1 in pulmonary hypertension and lung development

GLP-1 also has a beneficial effect on pulmonary hypertension. Activation of GLP-1 receptors in pulmonary arteries leads to vasodilation (157, 158). GLP-1 also plays an important part in lung development, and Liraglutide improved lung function and development in pups suffering from intrauterine growth restriction caused by ACE2-Ang(1–7)-MasR (159).

## 13 Summary of pulmonary effects of GLP-1

**Figure 10:**
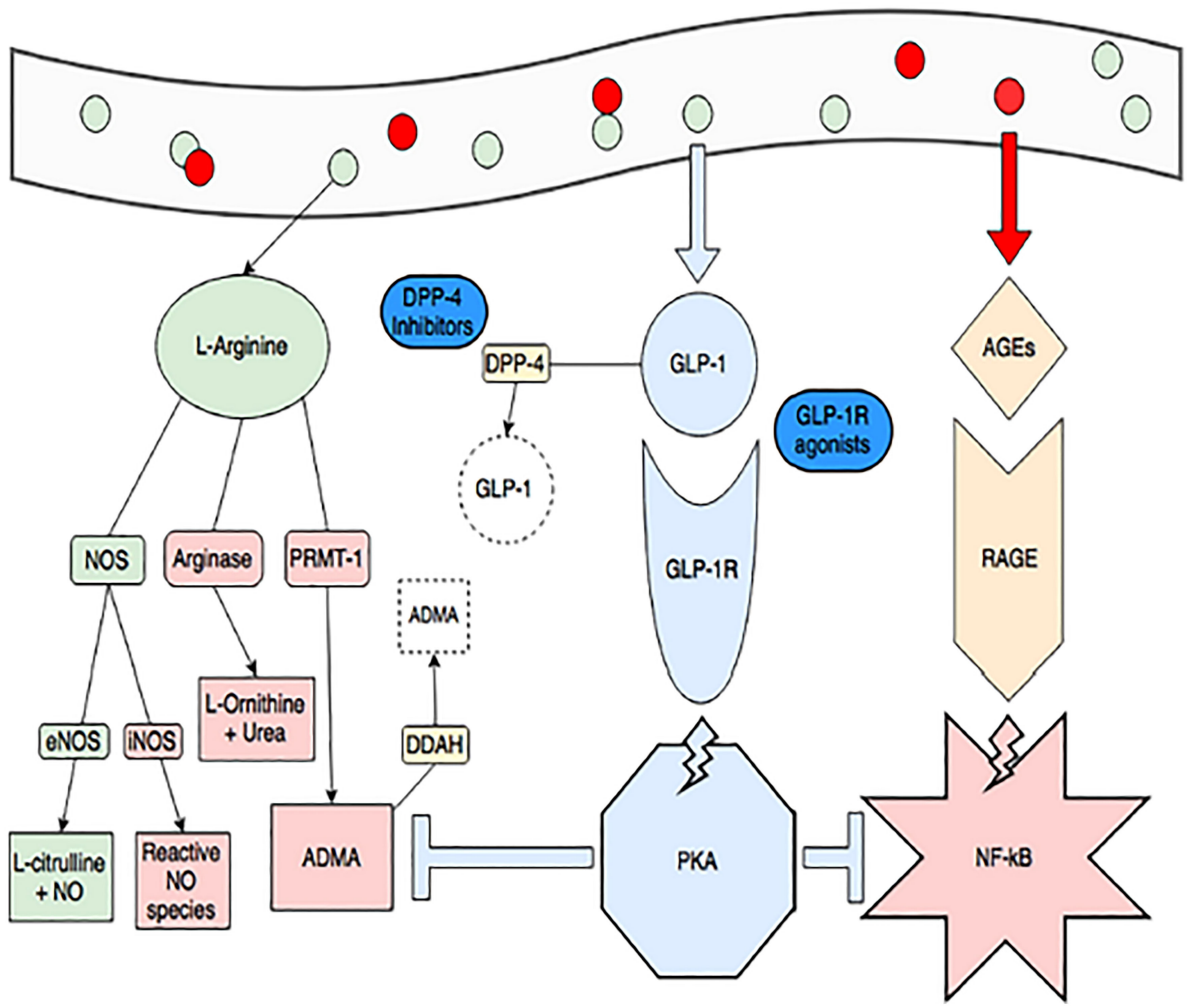
Obesity and consumption of foods high in advanced glycation end-products (red circles, AGEs) create a pro-inflammatory state through dysregulated arginine metabolism (increasing arginase activity and production of ADMA, in red) and activating RAGE-mediated, NF-kB inflammation (pink star). ADMA also inhibits endothelial NOS (eNOS) and increases NF-kB activity. GLP-1 production is spurred by consumption of L-arginine (green circles) and when it binds its receptor, it activates protein kinase A (blue octagon). This activity blunts RAGE-mediated inflammation and production of ADMA (blue T-lines). The GLP-1 pathway is also a target of treatments for diabetes and obesity. GLP-1 is rapidly degraded by DPP-4, and DPP-4 inhibitors (gliptins) are used to increase GLP-1. GLP-1 receptor agonists (exenatide and liraglutide) are also available (148). Figure modified from article 147 Open access (Journal of Immunology Research) permissible to re-use under a CC-BY 4.0 license).

**Figure 11:**
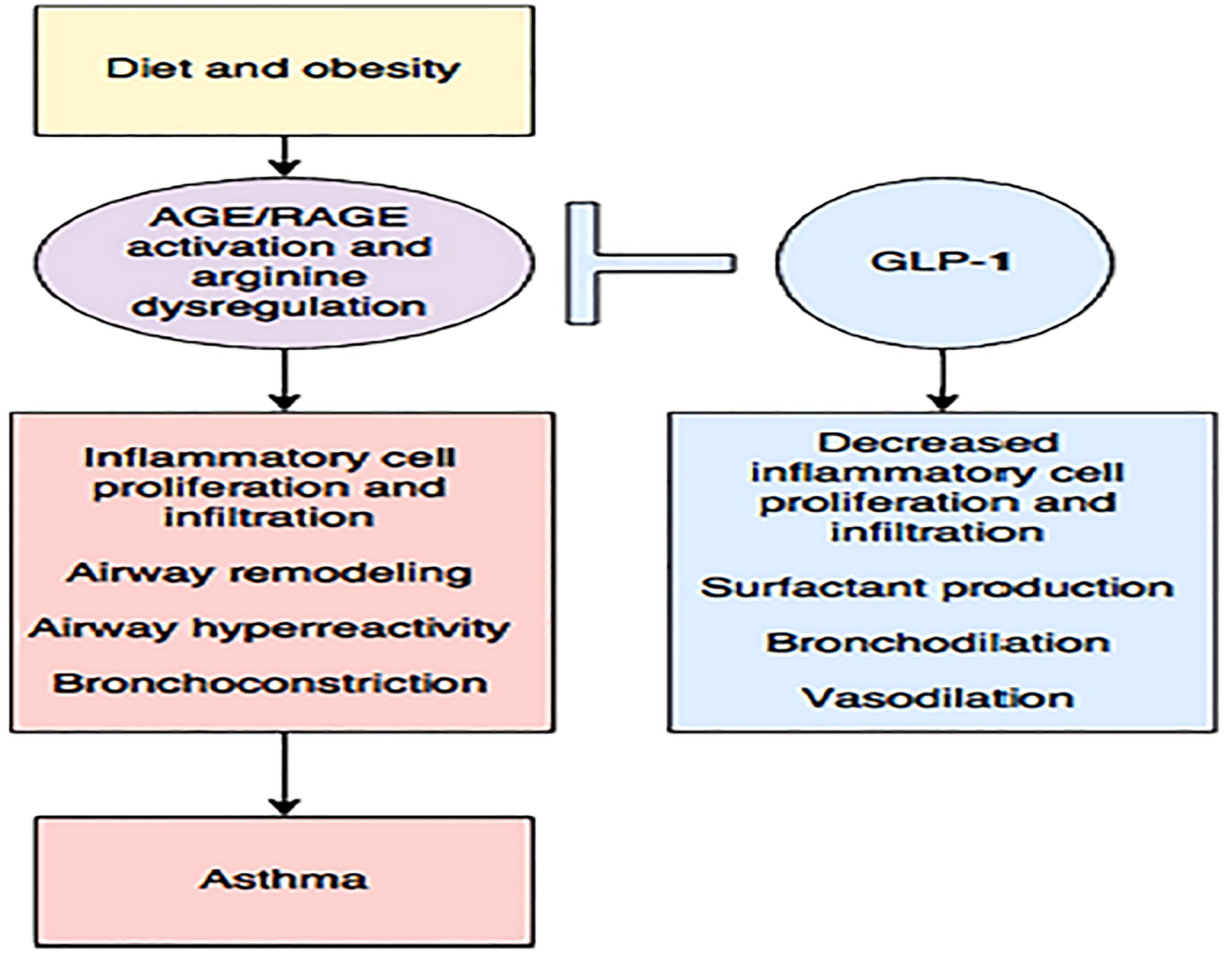
Diet and obesity may lead to dysregulated arginine metabolism and increase the production of advanced glycation end products (AGE) with subsequent activation of their receptor (RAGE), contributing to inflammation and asthma. The enhancing GLP-1 pathway may be the key to reducing this inflammation (148). Figure modified from articles 147 (Figure from Open access (Journal of Immunology Research) permissible to re-use under a CC-BY 4.0 license).

GLP-1 and its analogs are potentially beneficial in the respiratory system at most stages in life. (**Figures 10**, **11**) (148).

### 13.1 Renal system

Renal inflammation is a primary cause of kidney failure. Repeated kidney injuries ultimately result in end-stage renal disease. Diabetes is one cause of kidney damage. How diabetes causes inflammation is controversial, but it is known to promote the problem in both the organ and the whole body (160). Inflammatory cells, cytokines, and profibrotic growth factors cause vascular inflammation and fibrosis in diabetic nephropathy (DN). GLP-1, through its anti-inflammatory effects, reduces inflammation and fibrosis in diabetes (42).

The presence of oxidative stress in diabetic kidneys is a significant element in the inflammatory process. Oxidant/antioxidant imbalances activate NF-kB (161). GLP-1 receptor knockout mice have increased glomerular superoxide, upregulated renal NAD(P)H oxidase, and reduced renal cAMP and PKA activity. These changes lead to renal pathology. Activation of the cyclic adenosine monophosphate–protein kinase A (cAMP–PKA) pathway halts the synthesis of reactive oxygen species. GLP-1 receptor agonists activate cAMP-PKA pathway and protect against oxidative stress. Liraglutide reduced NADPH oxidase activity and increased cAMP-PKA activity in mice. It also enhanced glomerular hyperfiltration by improving glomerular nitric oxide and decreasing mesangial expansion (162, 163).

Advanced glycation end products are a common pathogenic stimulant in diabetes. They increase production of reactive oxygen species. GLP-1 agonists interfere with the signaling of receptors for advanced glycation end products. This leads to less oxidative stress and promotes protection against diabetic nephropathy (164).

Reactive oxygen species (ROS) increase the synthesis of monocyte chemotactic protein-1 (MCP-1) in diabetes (165). Increased NF-kB expression leads to higher levels of MCP-1, IL-1, and TNF-α. Macrophage activation generates a proinflammatory condition that causes structural damage to the kidneys. In the kidneys, prostaglandins serve a protective function. PGE2 synthesis is inhibited when macrophages secrete IL-1 and TNF-α. Reduced PGE2 levels hasten the inflammatory process in the kidneys (166). In rats with STZ-induced diabetes, exendin-4 decreases proteinuria and serum creatinine levels, and inhibits mesangial matrix expansion. It also protects against glomerular hypertrophy, monocyte infiltration and by reducing TGF-β, ICAM1, and CD14 in the renal cortex. Diabetes caused several histological changes in the renal tissue in another STZ-induced diabetes mouse model, including decreased height and continuity of the tubular brush border, vacuolization of proximal and distal tubular cells, necrosis of tubular and glomerular cells, hemorrhage, and mononuclear cell infiltration. Exendin-4 therapy resulted in a substantial reduction in all these lesions (167). In another similar mouse model, liraglutide resulted in restoration of catalase and glutathione peroxidase-3 levels, enzymes crucial in tissue protection against oxidative damage in kidneys (168).

GLP-1 protects diabetic kidneys. It lowers glucose levels and reduces inflammatory responses. GLP-1 receptor levels increase early in sepsis suggesting that it may have a protective role in this disorder as well (169). The use of recombinant human GLP-1 decreases the albumin content of the urine. In tubular tissue and human proximal tubular cells, it also reduces the production of multiple profibrotic factors including collagen I, alpha smooth muscle actin (SMA), fibronectin, and inflammatory proteins MCP-1 and TNF (HK-2 cells). Furthermore, in both diabetic tubular tissue and HK-2 cells, rhGLP-1 strongly decreased the phosphorylation of NF-kB and MAPK (170).

Sitagliptin inhibits inflammation and apoptosis. Use of sitagliptin in mice has been shown to decrease urine microalbumin, serum creatinine, blood glucose and blood urea nitrogen. It also decreased TNF-α receptor microRNA levels (171).

### 13.2 Skin

#### 13.2.1 GLP-1 effects on wound healing

Along with its anti-inflammatory role in other organs, GLP-1 agonists play a vital role in wound healing. During normal wound healing, fibroblasts secrete collagen and multiple cytokines to regulate the process. They also produce matrix metalloproteinases [MMP] and tissue inhibitors of matrix metalloproteinases [TIMP] (172)]. Matrix metalloproteinases promote degradation of extracellular matrix proteins (173). At high levels in wounds, MMPs delay wound healing (174, 175). This activity of MMPs is modulated by the tissue inhibitors of matrix metalloproteinases (TIMPs) (176). Increased activity of TIMPs is associated with better wound healing (177).

**Figure 12:**
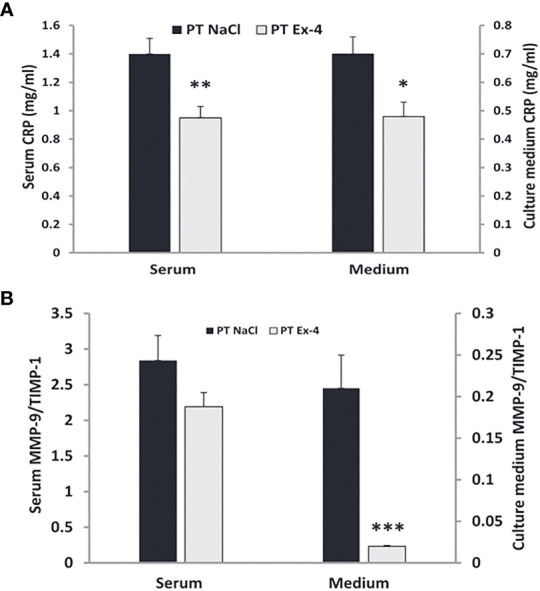
Use of GLP-1 Exendin-4 results in a) normalizes CRP levels in serum and medium b) low levels of MMP-9 and low MMP-9/TIMP ratios in serum and medium. **(A)** Data represent the mean ± SEM. *P<.05; **P<0.001, **(B)** Data represent the mean ± SEM. *P<.05; **P<0.01; ***P< 0.001 (182). Figure modified from article 183. Figure from Open access (European journal of pharmacology) permissible to re-use under a CC-BY 4.0 license).

Chronic skin wounds in patients with diabetes have high levels of MMP-9 and low levels of TIMPs with resultant high MMP-9/TIMP ratios (178). Higher MMP-9/TIMP ratios can also be seen in the serum of these patients (178, 179). C-reactive protein, another indicator of inflammation, is elevated in patients with foot ulcers (180). Use of exendin-4 in patients with chronic diabetic wounds normalizes CRP levels in serum and medium (181) (**Figure 12**). Low MMP-9/TIMP ratios are associated with earlier wound healing and better overall outcomes (183). Use of GLP-1, both *in vitro* and in-vivo, results in low levels of MMP-9 and low MMP-9/TIMP ratios in serum and medium, leading to quicker wound healing (182) (**Figure 12**).

#### 13.2.2 Psoriasis

Along with effects in wound healing, GLP-1 agonists result in an improvement in psoriasis symptoms (184). Psoriasis is an inflammatory condition that is associated with excessive secretion of proinflammatory cytokines (IL-2, IL-6, IL-8, IL-12, IL-19, IL-22, IL-23, IFN-γ and TNF-α) into blood as well as dermal tissue (185). Skin biopsies show up-regulation of GLP-1 receptors in psoriasis lesions (186).

Obesity is an inflammatory disorder with dysregulated innate immune responses that cause the number of natural killer cells in the circulation to decrease (187). Obesity, along with psoriasis, is associated with chronic systemic inflammation (188). There is also a well-documented positive correlation between obesity and psoriasis (189). Treating obesity with GLP-1 agonists results in improvement of glucose tolerance and an improvement in psoriasis. The studies completed to date have shown decreases in both psoriasis area and severity index (PASI) (179, 180, 190–192). Histopathological examination of psoriasis skin lesions after 12 weeks of liraglutide showed reduced epidermal thickness as well as evidence for lesion resolution (190). A plausible mechanism through which GLP-1 improves psoriasis is by blocking expression of IL-17, IL-22, IL-23 and TNF-α through the IL-23/Th-17 pathway (191).

## 14 Type 2 diabetes and other metabolic disorders

### 14.1 Diabetes and metabolic syndrome

Chronic hyperglycemia (high blood sugar) in diabetes can lead to the production of advanced glycation end products (AGEs) and reactive oxygen species (ROS), which can damage cells all throughout the body. Cardiovascular disease, renal failure, and neuropathy are a few possible effects of this ongoing low-grade inflammation. The metabolic syndrome, on the other hand, is a combination of diseases that also cause chronic inflammation, including obesity, hypertension, diabetes, excessive blood sugar, and abnormal cholesterol levels. Inflammatory chemicals like cytokines can be produced by adipose tissue in obese people, which can increase metabolic dysfunction and lead to insulin resistance (193).

#### 14.1.1 Liraglutide

Liraglutide decreased TNF-α, IkB, TLR2, and TLR4 mRNAs in peripheral blood mononuclear cells. Liraglutide appears to improve the metabolic profiles of obese type 2 diabetic patients and increase Sirtuin 1(SIRT1) expression, which in turn appears to suppress the pro-inflammatory NF-kB pathway. It also has an anti-inflammatory effect on vascular endothelial cells by increasing the generation of nitric oxide (193).

A sub-study of a randomized trial involving 54 with type 2 diabetes mellitus patients treated for 26 weeks with liraglutide or placebo examined whether liraglutide exerted anti-inflammatory effects through modulation of inflammatory gene expression in peripheral blood mononuclear cells (PBMCs) and Human monocytic cell line (THP-1) cells. When compared to baseline, the results showed that liraglutide dramatically lowered the production of TNF-A IL1B and raised CCL5 in PBMCs. The placebo group did not show these effects. THP-1 cells were used in an *in vitro* test to investigate the potential direct effects of GLP-1 receptor activation on inflammatory genes. The production of inflammatory genes by THP-1 cells was induced by LPS in the presence or absence of 2.5 nM recombinant GLP-1. GLP-1 did not influence any of the tested genes, suggesting that it has no direct effects on monocytes. GLP-1R is not expressed at the mRNA level in type 2 diabetes mellitus patients’ PBMCs or THP-1 monocytes. These results suggest that the effect of GLP-1 agonists on PBMCs are likely to be a secondary to changes in other tissues and/or the result of phenotypic alterations like weight loss or better glycemic control (194).

#### 14.1.2 Exenatide

HbA1c and blood sugar levels are considerably lowered by each GLP-1 receptor agonist. One of the GLP-1RAs is exenatide, a synthetic GLP-1RA made from exendin-4. Exenatide 2 mg administered once weekly (QW) has been demonstrated to significantly reduce fasting plasma glucose levels. Current formulations of exenatide, a GLP-1RA based on exendin, include twice daily (BID), a long-acting GLP-1RA, and once weekly (QW), a short-acting GLP-1RA. According to the DURATION research program, exenatide 2 mg QW has shown clinical efficacy and safety in persons with type 2 diabetes (T2DM). Exenatide QW has been demonstrated to reduce HbA1c more than exenatide BID because it requires fewer injections and has higher treatment compliance (195).

#### 14.1.3 Semaglutide

The Food Drug Administration (FDA) has approved the injectable GLP-1 receptor agonist semaglutide for use in the treatment of type 2 diabetes. The mean glycated hemoglobin level reductions in trials involving semaglutide patients have been reported to be as high as 1.8 percentage points, while the mean weight reductions have been reported to be as high as 6.5 kg (196).

#### 14.1.4 Tirzepatide

The once-weekly dual glucose-dependent insulinotropic polypeptide-GLP-1 receptor agonist tirzepatide outperformed the selective GLP-1 receptor agonist semaglutide in patients with type 2 diabetes who were taking metformin monotherapy, according to the SURPASS trial (196).

### 14.2 GLP-1 and polycystic ovary syndrome

PCOS is associated with hyperinsulinemia and a decrease in circulating levels of GLP-1 and GIP, two incretin hormones. Administration of either of the two improves insulin sensitivity and glucose metabolism in patients with PCOS (197).

#### 14.2.1 Liraglutide

In postmenopausal PCOS rat models, the effects of liraglutide on the cardiometabolic profile, the intrarenal renin-angiotensin system (RAS), and the blood pressure (BP) were investigated. Four-week-old female mice were treated with dihydrotestosterone (DHT) for 17 months and a placebo. Liraglutide was administered to postmenopausal PCOS rats over the last three weeks; and this resulted in a greater decrease in body weight, fat mass, food consumption, and insulin resistance than in control rats. Liraglutide improved both dyslipidemia and leptin levels in postmenopausal PCOS rats. In the control group, Liraglutide, only decreased intrarenal RAS transiently while increasing heart rate and decreasing blood pressure. In PCOS rats, liraglutide increased heart rate but did not affect blood pressure. Enalapril, an inhibitor of the angiotensin-converting enzyme, eliminated the BP differences between PCOS and control rats. Liraglutide and enalapril co-administration further lowered blood pressure only in control rats. In summary, Liraglutide lowered a number of cardiometabolic risk factors in postmenopausal PCOS. Hyperandrogenemia, on the other hand, prevented Liraglutide from regulating blood pressure in postmenopausal PCOS. The stimulation of intrarenal RAS by androgens may contribute to BP increases in postmenopausal PCOS (198).

In a prospective observational study of the impact of liraglutide on weight loss in obese and overweight people with PCOS, 84 obese women with PCOS were given daily subcutaneous injections of liraglutide beginning with a dose of 0.6 mg. The dose was increased to 1.2 mg and then 1.8 mg if the compound was well tolerated. The treatment lasted 4 weeks and subjects were monitored for a total of 27 weeks. They had a significant decrease in weight and BMI. Weight and atherothrombotic markers, such as endothelial function and clotting time, significantly decreased in obese women with PCOS who were given liraglutide (1.8 mg per day) vs those given placebo. In another study, liraglutide (1.8 mg) had a beneficial effect on body weight, quality of life (QOL) and depression (197, 198).

In a randomized control trial, liraglutide and a placebo were given to 72 PCOS, BMI>25, insulin-resistant women for 26 weeks. Liver fat concentration, the prevalence of nonalcoholic fatty liver disease (NAFLD), and visceral adipose tissue (VAT) were investigated. DXA dual-x-ray absorptiometry was used to measure body composition, Proton magnetic resonance spectroscopy (1H-MRS) to measure liver fat content, MRI to measure VAT (Visceral adipose tissue) and an oral glucose tolerance test to measure glucose metabolism. In comparison to placebo, liraglutide treatment reduced the prevalence of NAFLD by two-thirds, the amount of fat in the liver by 44%, visceral adipose tissue by 18%, and body weight by 5.2 kg (5.6%) (199).

#### 14.2.2 Exenatide

A randomized single-blinded trial was conducted in 119 PCOS women without diabetes and a BMI of 30 to 45 mm/kg. Exenatide (EXE) (2 mg weekly), Dapagliflozin (DAPA) (10 mg daily), Exenatide + Dapagliflozin (2 mg weekly/10 mg daily), Dapagliflozin (10 mg) + Metformin (MET) (2000 mg extended release daily), or Phentermine (7.5 mg)/Topiramate were given to the patients for 24 weeks (46 mg extended release daily). All medications caused decreases in fasting blood sugar, testosterone, and blood pressure. Both combinations of Exenatide, Dapagliflozin plus Phentermine, Topiramate resulted in significant weight loss and waist circumference decrease. Exenatide plus dapagliflozin was the only treatment that significantly decreased (fasting) blood sugar and improved insulin sensitivity. This combination, therefore, outperformed others in terms of clinical and metabolic effects (200).

For a period of 12 weeks, a combination of exenatide plus metformin and metformin monotherapy was assessed in fifty obese/overweight women of reproductive age. Forty patients completed the study. In terms of lowering weight, body mass index (BMI), and waist circumference, combination treatment outperformed metformin monotherapy. Additionally, with combination therapy as opposed to metformin, showed lower levels of fasting glucose, oral glucose tolerance test (OGTT) 2-h glucose, and OGTT 2-h insulin. Thus, in overweight/obese women with PCOS, combination treatment is more effective than metformin alone by improving insulin sensitivity, with tolerable short-term side effects (201).

A meta-analysis of the effects of insulin sensitizers in PCOS patients showed that GLP-1 receptor agonists are superior to metformin in improving insulin sensitivity, and metformin is superior to thiazolidinediones in decreasing BMI. A combination of GLP-1 receptor agonists and metformin had little effect on menstrual frequency or serum testosterone. Metformin combined with thiazolidinediones were particularly effective in promoting the recovery of menstruation in PCOS patients. A combination of GLP-1 receptor agonists and metformin or thiazolidinediones was superior to metformin monotherapy as a treatment for hyperandrogenism (202, 203).

### 14.3 Obesity

Between 1960 and 1980, obesity prevalence among adults aged 20-49 was between 13% and 15% (204). An estimated 33.0% of American people aged 20 and above are overweight, 35.7% are obese, and 6.3% are severely obese, according to data from the 2009–2010 National Health and Nutrition Examination Survey (NHANES), which used measured heights and weights (205). In the United States between 2017 and 2018 adults aged 20 and above had a prevalence of obesity of 42.4% and a prevalence of severe obesity of 9.2% (206). At present 33% of US adults are overweight and are in the 40-59 age group. Further weight gain is predicted by 2030 (205, 206). Men and women had equal obesity prevalence rates overall, but women were more likely to have severe obesity than men. Non-Hispanic black men and women had the highest prevalence of severe obesity, and non-Hispanic Asian adults had the lowest (206). In June 2013, the American medical association first declared obesity a disease. Obesity is now the most prevalent chronic disease in the United States; it results in $147 billion in health care spending annually (204). GLP-1 receptor agonists decrease appetite, increase satiety, reduce food intake and decrease weight gain (207).

#### 14.3.1 Liraglutide

The GLP-1 receptor agonist Liraglutide may only decrease appetite for a short period of time. After 10 days of treatment with Liraglutide, patients had decreased responses in the insula and putamen to food pictures vs the control group treated with insulin glargine. After 12 weeks of treatment, there were no differences between the groups. GLP agonists may initiate weight loss, but not maintain it (206).

Treatment with a GLP-1R agonist resulted in a greater weight loss than control treatment did. The GLP-1R agonist had beneficial effects on systolic and diastolic blood pressure, plasma concentrations of cholesterol, and glycemic control, but did not have a significant impact on plasma concentrations of liver enzymes. Taking the GLP-1R agonist was associated with nausea, diarrhea, and vomiting, but not with hypoglycemia (208).

Liraglutide 3.0 mg (Saxenda^®^; Novo Nordisk), as an adjunct to a caloric restriction and increased physical activity, has been approved for weight management in the USA and Europe. The Satiety and Clinical Adiposity Liraglutide Evidence (SCALE) Phase III trial in non-diabetic and diabetic people investigated the safety and efficacy of liraglutide 3.0 mg (once daily subcutaneous injections). In this weight management program subjects treated with liraglutide 3.0 mg experienced a dose-dependent weight loss ranging from 6.0 kg to 8.8 kg, whereas subjects treated with placebo (on diet and exercise alone) had a mean weight loss of 0.2 kg to 3.0 k (209).

#### 14.3.2 Semaglutide

Semaglutide is a GLP-1 receptor agonist that is dosed once a week subcutaneously based on its extended half-life. The molecule binds strongly to albumin because of a large fatty acid chain attached to the lysine in position 26. A phase 2 dose-finding trial in subjects with type 2 diabetes showed clear dose-dependent effects on HbA1c and weight over 12 weeks of treatment. 1.6 mg/week resulted in an absolute weight loss of 4.82 kg compared to the placebo group’s 1.18 kg and a drop in HbA1c of up to 1.7%. As a secondary end-point, a direct comparison with liraglutide (up to 1.8 mg) was made. Semaglutide appeared to be more efficacious for weight loss than liraglutide (2.6 kg) (210).

Current trials: The significant phase 3 clinical program assessing the efficacy and safety of semaglutide (SUSTAIN) in type 2 diabetes has been completed. To avoid the requirement for subcutaneous injections and new formulations of semaglutide have been developed. An orally available product is in phase 3. This formulation is combined with the absorption enhancer SNAC (sodium N-[8-(2-hydroxy benzoyl)amino] caprylate), causing a localized increase in pH and enabling a higher solubility and protection from enzymatic degradation. According to this study, both hyperglycemia and hypoglycemia in patients with type 1 diabetes gives rise to endothelial dysfunction, oxidative stress, and inflammation and GLP-1 can be useful to counterbalance these effects. Thus, it supports the usefulness of GLP-1 and its analogs in the management of type 1 diabetes (210).

### 14.4 GLP-1 and type 1 diabetes mellitus

The role of GLP-1 in patients with type 2 diabetes is well-studied and well-established. Surprisingly, GLP-1 has been slow to emerge in patients with T1DM. The use of GLP-1 agonists may be considered in T1DM patients who are overweight or obese and not at glycemic goals. GLP-1 decreases inflammation in pancreas which can help in preserving beta cells and ameliorate the progression to Type 1 diabetes. Further studies are required to fully understand the role of GLP-1 in T1DM management (210).

### 14.5 SEPSIS

Sepsis is characterized by widespread inflammation and organ dysfunction. It continues to be a major cause of illness, disability, and death at all ages (211). Hormones in the body, such as oxytocin ghrelin, alpha MSH, ACTH and hCG, have a significant role in reducing the inflammatory response that occurs during sepsis (3–6). GLP-1 plays a crucial role in regulating the cytokine storm by binding to receptors in a wide variety of tissues including the brain, kidneys, liver, and lungs. It reduces proinflammatory processes and boosts anti-inflammatory ones throughout the body. Even though GLP-1’s use of sepsis has been encouraging in animal models, there have been no human trials. Additional research on the use of GLP-1 in patients with sepsis may further elucidate its anti-inflammatory properties and spur human studies.

## 15 Conclusion

GLP-1 and its agonists have opened new avenues for treatment of inflammatory diseases to mitigate organ dysfunction, septicemia, and post-sepsis syndrome. Further clinical research is required. Besides peptide hormones like ghrelin, oxytocin and hCG, consideration should be given to incretin and other related peptides.

## Author contributions

SM, SP, MA, DeL, PK, SA, FN and JR conceived of the article, compiled the literature, and interpreted its contents. DeL, HY, KT, MB and JR added critical intellectual content to the manuscript and can be considered experts on the topic. All authors provided critical feedback and helped shape the research and analysis. All authors contributed to the article and approved the submitted version.
